# Characterization and Otoprotective Effects of Polysaccharides from *Arthrospira platensis*

**DOI:** 10.3390/molecules30020224

**Published:** 2025-01-08

**Authors:** Matteo Banti, Mercedes Garcia-Gil, Lorenzo Guidotti, Graziano Di Giuseppe, Simona Rapposelli, Daniela Monti, Silvia Tampucci, Marinella De Leo, Francesca Gado, Paola Nieri, Clementina Manera

**Affiliations:** 1 Department of Pharmacy, University of Pisa, Via Bonanno, 6, 56126 Pisa, Italy; matteo.banti@phd.unipi.it (M.B.); simona.rapposelli@unipi.it (S.R.); daniela.monti@unipi.it (D.M.); silvia.tampucci@unipi.it (S.T.); marinella.deleo@unipi.it (M.D.L.); 2 Department of Biology, University of Pisa, Via Luca Ghini, 13, 56126 Pisa, Italy; mercedes.garcia@unipi.it (M.G.-G.); l.guidotti1@student.unisi.it (L.G.); graziano.di.giuseppe@unipi.it (G.D.G.); 3 Interdepartmental Center of Marine Pharmacology (MarinePHARMA), University of Pisa, 56126 Pisa, Italy; 4 Center for Instrument Sharing of the University of Pisa (CISUP), University of Pisa, Lungarno Pacinotti, 43/44, 56126 Pisa, Italy; 5 Department of Pharmaceutical Sciences, University of Milan, 20133 Milan, Italy; francesca.gado@unimi.it

**Keywords:** polysaccharides, microalgae, LC-MS, otoprotective effect, radical scavenging activity, *Arthrospira* (*Spirulina*) *platensis*

## Abstract

Hearing loss is one of the most common sensory disorders in humans, and a large number of cases are due to ear cell damage caused by ototoxic drugs including anticancer agents, such as cisplatin. The recent literature reported that hearing loss is promoted by an excessive generation of reactive oxygen species (ROS) in cochlea cells, which causes oxidative stress. Recently, polysaccharides from the cyanobacterium *Arthrospira platensis* showed many biological activities, including antioxidant activity, suggesting their potential use to combat hearing loss. On these bases, this study describes the extraction, purification, and characterization of water-soluble polysaccharides from *A. platensis* (SPPs) and the investigation of their protective role against cisplatin toxicity on House Ear Institute-Organ of Corti (HEI-OC1) cells. The results showed that SPPs (5–80 µg/mL) induced a dose-dependent increase in viability, statistically significant at 40 µg/mL and 80 µg/mL. Moreover, SPPs, evaluated at 80 µg/mL, inhibited the cisplatin-induced ROS level increase in HEI-OC1. This evidence highlights the potential of SPPs as natural candidates to protect cochlear ear cells against ototoxic oxidative agents. Moreover, in view of the potential use of microalgal polysaccharides to realize hydrogels, SPPs could also represent a healthy carrier for other topically administered otoprotective agents.

## 1. Introduction

Sensorineural hearing loss (SNHL) is the most common type of hearing loss caused by damage to the inner ear or auditory nerve, and a large number of cases are due to ear cell damage caused by ototoxic drugs. Indeed, drug-induced ototoxicity is a major side effect of many pharmacological treatments. About two hundred drugs are known to produce toxicity to sensory cells in the inner ear [1]. Among the most ototoxic drugs, cisplatin (cis-diamminedichloroplatinum II) and other platinum salt agents may induce serious consequence in the inner ear, i.e., tinnitus and permanent damage with a high-frequency of hearing impairment and outer ear cell loss [2]. Cisplatin-like compounds are powerful anticancer agents able to induce covalent bonds between the platinum atom and DNA purine bases inducing intra- and inter-strand crosslinks in DNA and then promoting apoptosis [3]. Mitochondrial DNA represents a vulnerable target of platinum-derived compounds. Platinated mitochondrial DNA induces excessive ROS formation and activates apoptotic cascades. Apoptosis and other cell death mechanisms may occur also in cochlear cells where cisplatin induces oxidative stress via a high amount of ROS production [4,5,6]. Different preclinical and clinical pharmacological strategies have been proposed involving the use of antioxidants that can protect cells against ROS generation [7], such as curcumin [8], vitamin E [8], *N*-acetylcysteine [9], dexamethasone [10], amifostine [11], and tertiary butyl hydroquinone [12]. Recently, Rybak and collaborators reviewed preclinical studies and also a few clinical trials with natural products to reduce/avoid hearing loss due to cisplatin therapy [13]. At present, there is no FDA- or EMA-approved treatment with demonstrated efficacy in preventing or reducing cisplatin-induced ototoxicity. Therefore, searching for non-toxic, natural, cost-effective antioxidant compounds is still relevant.

Recently, growing attention has focused on natural polysaccharides that play important functions as food and medicinal agents. Polysaccharides are essentially polymeric carbohydrates in which the monosaccharides are linked by glycosidic bonds. Many natural polysaccharides have recognized antioxidant properties [14,15], and, accordingly, their protective effects against the toxicity by cisplatin have been reported in different tissues [16,17,18,19,20]. Marine organisms represent a promising source of nutrients and bioactive compounds including polysaccharides. Numerous studies have shown that polysaccharides of marine origin have a variety of bioactivities [21,22], such as anti-coagulant, antimicrobial, antiviral, anti-inflammatory, anticancer, and antioxidant activities [23,24]. In recent years, particular attention has been paid to polysaccharides derived from microalgae which, compared to other sources, have proven to be safe, biocompatible, biodegradable, stable, and versatile [25,26,27]. Several lines of evidence have shown that microalgal polysaccharides possess the ability to prevent the accumulation of free radicals and reactive chemical species, thus acting as a protective system against these agents [28]. 

*Arthrospira platensis*, also commonly known as *Spirulina platensis* (SP), is a blue-green microalga that is successfully produced and commonly consumed as a nutraceutical food supplement [29]. It contains numerous valuable compounds such as lipids (6–13%), protein (60–70%), and polysaccharides (15–20%) [30]. Polysaccharides from SP (SPPs) gained much attention because of their high biological activities, including, anti-inflammatory, immune regulation, antiviral, and antioxidant activities [31,32,33,34,35,36].

The aim of this study was to investigate the in vitro antioxidant properties and the effects of isolated SPPs on cisplatin-induced ear cell damage. Our findings suggest that SPPs may represent a potential otoprotective agent for developing strategies to reduce cisplatin-induced ototoxicity.

## 2. Results and Discussion

### 2.1. Chemical and Spectral Analysis of SPPs

In the present study, the SPPs yield obtained by ultrasound-assisted extraction combined with hot water extraction was 3.7%, in accordance with the method reported by Wang et al. [37]. The proximate composition result confirmed the presence of carbohydrates, sulfate, and protein at levels of 80.5 ± 2.1%, 9.7 ± 0.9%, and 8.0 ± 0.7%, respectively. Similar results were also reported in the literature from various microalgae [25].

**Figure 1:**
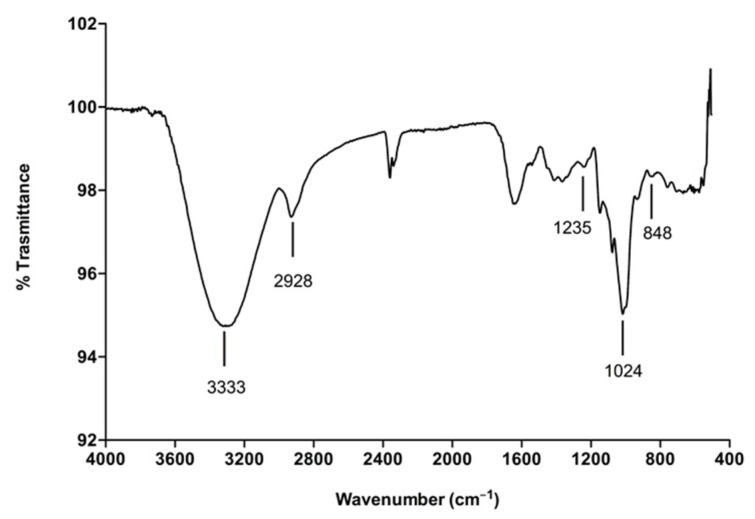
FT-IR analysis of SPPs isolated from *Spirulina platensis*.

FT-IR analysis of SPPs was performed by an infrared spectrophotometer, and the spectra concerning the band position and intensities are presented in Figure 1. The peaks at 3333 cm^−1^ and 2928 cm^−1^ are representative of the presence of hydroxyl and methylene groups [38]. The absorption peaks at 1600−1650 cm^−1^ are due to the bound water [39], and the bands in the region 1200−1000 cm^−1^ are typical for polysaccharides [40]. In particular, the peak at 1024 cm^−1^ indicates the presence of C6 fucose’s and galactose’s groups and the C-O stretching vibration of C-O-SO_3_ groups [41]. The sulfate groups in the backbone structure of SPPs are also confirmed by the presence of the peaks at 1235 cm^−1^ and 848 cm^−1^ that are assigned to the SO asymmetric stretching vibration (S=O) and COS stretching vibration (C-O-S) of sulfate at the C4 axial [42,43].

**Figure 2:**
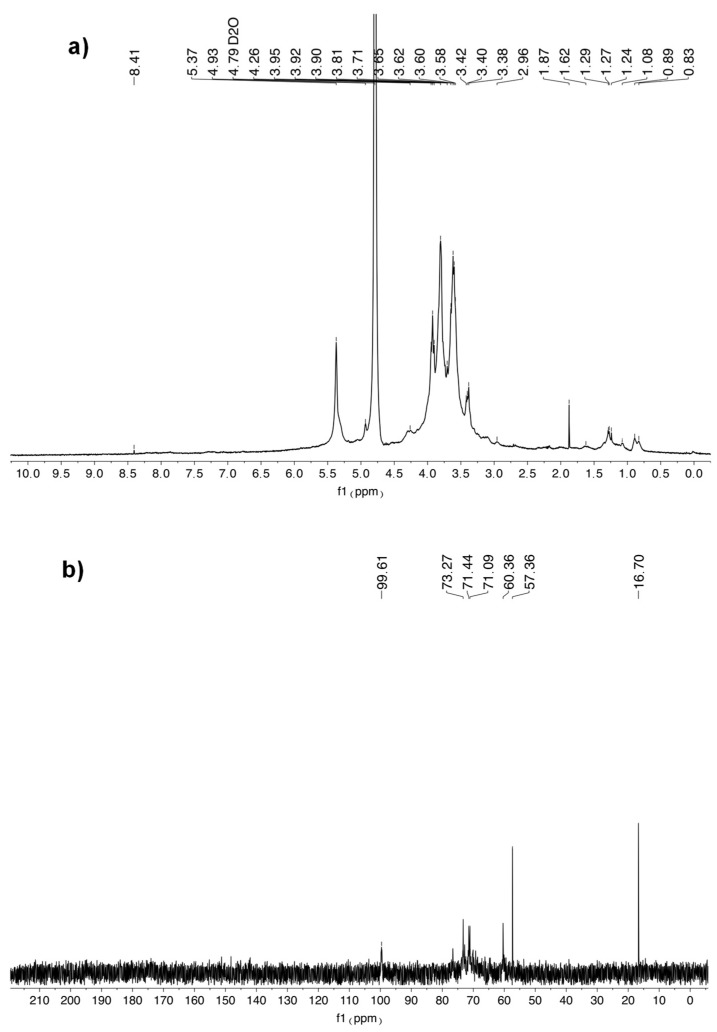
The ^1^H-NMR (**a**) and ^13^C-NMR (**b**) spectrum of SPPs.

The ^1^H-NMR spectrum (Figure 2) shows the typical signal of polysaccharides between 3 and 5 ppm [44]. The anomeric proton is recovered at 5.37–4.93 ppm [45]. The presence of residual peaks between 3 and 3.90 ppm indicated the presence of galactose, glucose, and 1,6-glucose sugar residues [43]. The signals at 60.36 ppm in the ^13^C-NMR spectrum indicated the presence of β-glucan (Figure 2) [43]. The presence of α-acetyl-glucosamine is confirmed by the correlation between the ^1^H-NMR and ^13^C-NMR (71.44, 57.36/3.95, and 4.27 ppm) [43]. Moreover, the correlation peaks at 99.61 and 4.27 ppm (C/H) denoted the presence of α-d-glucose residues. The reported structural evidence is in accord with the earlier studies of polysaccharides isolated from other algae [45,46].

### 2.2. Monosaccharide Composition 

**Figure 3:**
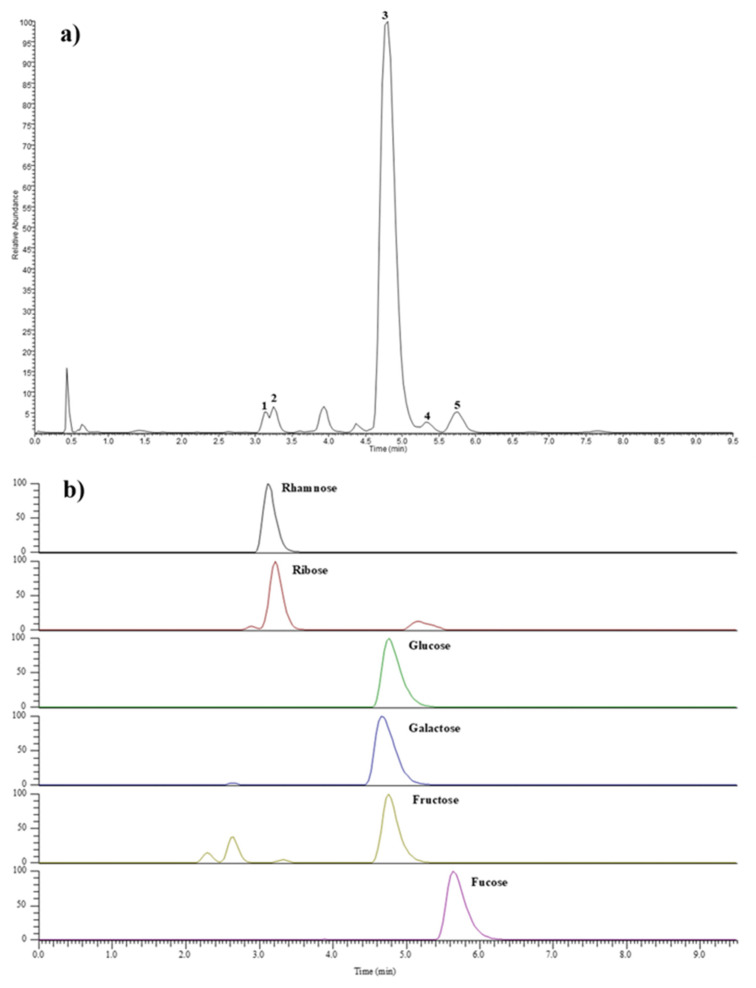
LC-MS profile registered in negative ionization mode of 1-phenyl-3-methyl-5-pyrazolone (PMP)-labeled monosaccharides of the hydrolyzed SPPs (**a**) and standard monosaccharides (**b**).

The identification of sugar components was carried out by comparison of their retention time (t_R_), UV absorption, full mass spectra, and mass fragmentation pattern with the literature data [47] and data obtained by injection of monosaccharide standards after derivatization with PMP in the same conditions. A mass error < 5 ppm was considered for compound attribution. The LC-MS chromatogram registered in negative ionization mode is shown in Figure 3, while chromatographic and spectral data in both +ESI and −ESI mode are reported in Table 1. The main peak at t_R_ = 4.8 min (peak 3), showing a deprotonated molecular ion [M − H]^−^ at *m*/*z* 509.2043, was attributed to a hexose-PMP, but it was not possible to discriminate among glucose, galactose, and fructose due to the coelution of the three monosaccharides having very close t_R_ values and identical mass spectral data. However, based on NMR experiments, glucose and galactose are good candidates as constituents of SPPs, according to previous studies [37,43]. Other minor peaks were identified as rhamnose (peak 1), ribose (peak 2), and fucose (peak 5), as confirmed by chromatographic and MS data of pure injected standards. Finally, peak 4 was annotated as a pentose, as deduced by the presence in the full MS of a deprotonated molecular ion [M − H]^−^ at *m*/*z* 479.1940 corresponding to the PMP-derivative and product ions at *m*/*z* 215.08, 173.07, and 92.05, as reported for the previous PMP-monosaccharides. The injection of xylose-PMP and arabinose-PMP as reference pure compounds led to exclusion of the presence of both sugars; thus, peak 4 remained unidentified. Based on these results, polysaccharides from *S. platensis* analyzed in this study were mainly composed of glucose/galactose, together with rhamnose, ribose, and fucose.

### 2.3. Radical Scavenging Activity

The antioxidant activity of polysaccharides from SPPs has been demonstrated by using multifarious free radical assays and/or by enhancing antioxidant enzyme activity [36]. Moreover, it is also suggested that the content of sulfated polysaccharides is related to the antioxidant activity of polysaccharides derived from microalgae [28]. As reported in the literature, it is possible that the sulfate groups increase the hydrogen release capacity of the anomeric carbon, increasing the hydrogen supply capacity of the polysaccharide and, therefore, determining its antioxidant activity [28]. In addition, sulfate groups produce an acidic environment that promotes electrostatic trapping of free radicals [48].

In order to investigate the radical scavenging properties of our isolated SPPs, hydroxyl radical (^•^OH scavenging and the ABTS radical (ABTS^•^) assays at different concentrations of SPPs were studied by following the standard methods. 

^•^OH in cells can easily react with many biological macromolecules such as proteins and nucleic acids and cause tissue damage and cell death. Thus, removing ^•^OH is very important for reducing or eliminating the damage induced by ^•^OH in food and pharmaceutical applications and, thus, for the protection of living systems [49]. 

The ABTS radical (ABTS^•^) is sensitive towards most antioxidants, and its decolorization reveals the capacity of an antioxidant species to donate electrons or hydrogen atoms to deactivate the radical species. Currently, this method is widely used to evaluate the radical scavenging activities of natural products [50]. 

**Figure 4:**
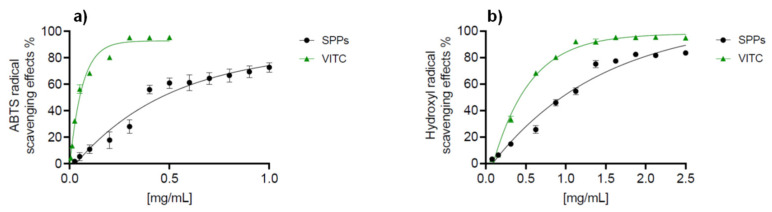
Radical scavenging activities of SPPs (curve of inhibition percentage versus SPP concentration). (**a**) ABTS radical scavenging activity (ABTS^•^ assay); (**b**) hydroxyl radical scavenging activity (^•^OH assay). VITC, ascorbic acid. Data are means ± SEM from 3 experiments.

As shown in Figure 4, the trend in ^•^OH and ABTS^•^ scavenging activities of SPPs gradually improved as their concentrations increased. The percentage of inhibition in both assays are expressed using effective concentration (EC50) values, which are reported as the amount of antioxidants (SPPs) required to decrease the initial concentration of scavenging radicals by 50%. 

The results demonstrated higher activity in the ABTS^•^ scavenging assay than in the ^•^OH one, with an EC50 of 0.328 mg/mL and 0.926 mg/mL, respectively. These values are in accordance with those reported in the literature for polysaccharides from *Spirulina platensis* [28,36]. In addition, the radical scavenging activity in both cases was lower than that of the ascorbic acid (VITC) used as a positive control.

### 2.4. Effect of SPPs on Viability of HEI-OC1 Cells

**Figure 5:**
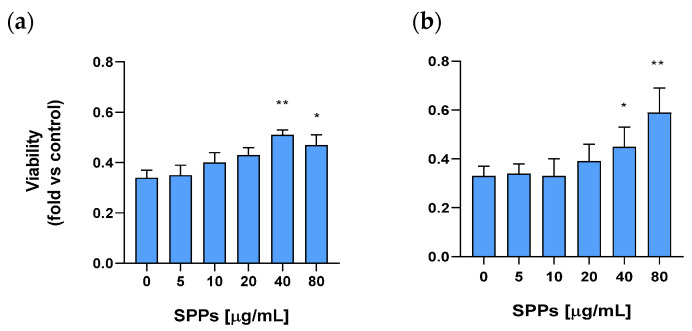
(**a**) Effect of SPPs on viability of HEI-OC1 cells. Data were normalized to the absorbance of the cells grown in the absence of SPPs or their solvent; (**b**) effect of preincubation with SPPs on viability of HEI-OC1 cells treated with cisplatin. Data were normalized to the absorbance of the cells grown in the absence of SPPs and their solvent. Data are presented as means ± SEM from 3–4 experiments. Significance was measured using a non-parametric ANOVA followed by Tukey’s multiple comparisons test. * *p* < 0.05, ** *p* < 0.01 vs. cells cultured in the absence of SPPs.

HEI-OC1 cells, a well-known in vitro model to screen ototoxic drugs [51], were grown in the presence of different concentrations of SPPs (5–80 µg/mL) for 48 h in the presence or the absence of cisplatin 5 μM. Viability was measured with the MTT assay as described in the Methods section). Cisplatin decreased viability by about 70% compared to control (Figure 5 at concentration “0” of SPPs). When SPPs were added in the presence of cisplatin, they increased viability in a dose-dependent manner from 10 to 80 µg/mL, reaching the maximal increase at 40 µg/mL. (Figure 5a). 

In order to clarify whether the effect of the SPPs was due to their binding to cisplatin and an inhibition of its transport inside the cell, in a set of experiments, cells were preincubated in the presence of different concentrations of SPPs (from 5 to 80 µg/mL) for 24 h, and then SPPs were removed via washing once with phosphate buffer saline. Then, cells were incubated with cisplatin in the absence of SPPs, and viability was measured 48 h after this addition. As shown in Figure 5b, preincubation with SPPs followed by treatment with cisplatin induced a dose-dependent increase in viability. The increase after preincubation with 40 and 80 µg/mL polysaccharides was statistically significant and reached the maximum at 80 µg/mL (Figure 5b).

### 2.5. SPPs Significantly Reduced the Cisplatin-Induced ROS Level Increase in HEI-OC1 Cells

To investigate the effects of the SPPs on intracellular ROS generation induced by cisplatin, HEI-OC1 cells were treated with 30 µM cisplatin in the presence or the absence of different concentrations of SPPs for 4 h and incubated with 5 µM 2′-7′-dichlorodihydrofluorescein diacetate (DCFH-DA) as a marker of ROS generation. The different concentration and time exposure of cisplatin (30 µM, 4 h) in comparison to the experiments described in Section 2.4 (5 µM, 48 h) is due to the need for working in a condition excluding great death of cells since ROS measurement is greatly linked to cell viability. 

**Figure 6:**
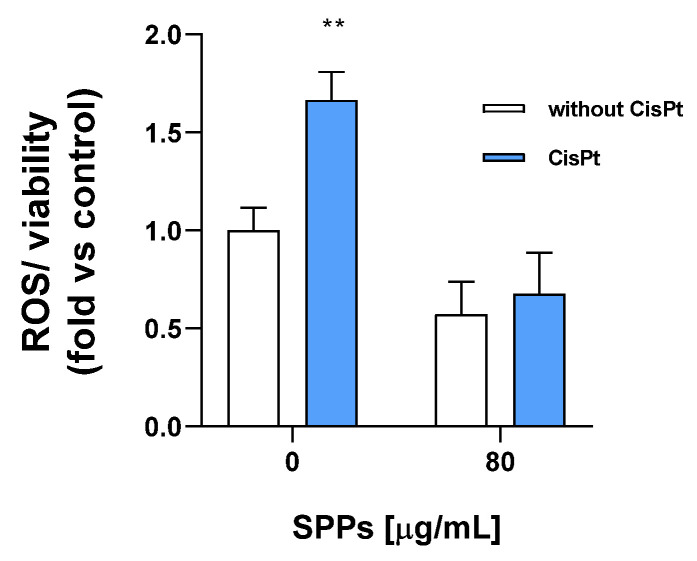
HEI-OC1 cells were treated with 30 µM cisplatin in the presence or the absence of SPPs. ROS levels were normalized to the values from cells grown in the absence of SPPs and their solvent. The data are expressed as means ± SEM of 3 experiments. Statistical significance obtained with Student’s *t*-test: ** *p* < 0.01 vs. control (correspondent blank bar). CisPt, cisplatin.

In any case, for the same reason just mentioned, we have analyzed the data normalizing against cell viability (ROS/viability, see Y axis Figure 6) to obtain a more standardized value. 

Under the above condition, cisplatin significantly increased ROS generation (1.67  ±  0.25-fold compared to control) only in the absence of SPPs, while no significant difference was observed between the two groups when SPPs at 80 µg/mL were added (Figure 6).

## 3. Materials and Methods

### 3.1. Materials 

*Arthrospira platensis* (*Spirulina platensis*) was purchased from Spirufarm S.r.l. (Casalbuttano ed Uniti, Cremona, Italy). Ethanol 95%, acetone, diethyl ether, hydrochloric water acid solution 0.5 N, sodium sulfate, barium chloride, galactose, sulfuric acid 95.0–98.0%, trifluoroacetic acid (TFA), the Lowry assay kit, and papaine were purchased from Merck Life Science S.r.l. (Milan, Italy). Methanol, water, and formic acid used for ultra-high-performance chromatography (UHPLC) were purchased from Merck KGaA (Darmstadt, Germany). House Ear Institute-Organ of Corti 1 cells (HEI-OC1) were a gift from Prof. Federico Kalinec (UCLA, University of California Los Angeles, Los Angeles, CA, USA).

### 3.2. Extraction, Purification, and Proximate Composition Analysis of SPPs

The SPPs were obtained by ultrasound-assisted extraction combined with hot water extraction according to the Wang et al. method [37]. Briefly, Arthrospira platenis powder (8 g) was added to distilled water (320 mL). The mixture was ultrasonicated for 1 h (25 °C, 120 Watt, VWR^®^ USC 200T, Leicestershire, UK). Subsequently, the mixture was vigorously stirred and heated at 80 °C for 8 h, followed by centrifugation (1220 RCF, 30 min). The supernatant was collected and kept at 80 °C until the volume became 1/5 of the original volume. Then, cold 95% ethanol was added at a volume 5 times that of the extract. The ethanol mixture was placed at 4 °C overnight, and afterwards, it was centrifuged (1220 RCF for 20 min). The precipitate was triturated with acetone, collected by filtration, and then dried to obtain 5.67 g of crude polysaccharides.

The crude polysaccharides were then dissolved in distilled water (1:14), adjusted to pH 7.0 by hydrochloric acid, added with papain (3% of the crude polysaccharides), and then heated at 50 °C for 2.5 h. Then, the enzyme was inactivated by boiling the mixture for a few minutes, and the mixture was treated with 5% of trichloroacetic acid and kept overnight at 4 °C. The mixture was centrifuged to obtain the supernatant, which was adjusted to pH 8.0 by concentrated ammonia, followed by the addition of H_2_O_2_ solution (30%) which was kept a 5% the final concentration and stirred for 2 h at 55 °C. Subsequently, five times the volume of 95% ethanol was added and stored at 4 °C overnight, and afterwards, the mixture was centrifuged (1220 RCF for 20 min). The final precipitate was washed with acetone and diethyl ether, collected by filtration, and then dried. The yield percentage was calculated using the following formula.

$$\mathrm{Yield}\;(\%)=\frac{\;\mathrm{Dry}\;\mathrm{weight}\;\mathrm{of}\;\mathrm{the}\;\mathrm{SPPs}\;}{\;\mathrm{Dry}\;\mathrm{weight}\;\mathrm{of}\;\mathrm{the}\;\mathrm{algae}\;\mathrm{sample}\;}\times 100$$

The total carbohydrate, sulfate, and eventual protein residue in the SPP extract were estimated by the phenol-sulfuric acid, gelatin–barium, and Lowry methods, respectively [52,53,54]. In these analyses, glucose, sodium sulfate, and bovine serum albumin were used as standards, for carbohydrate, sulfate, and protein content evaluation, respectively.

### 3.3. FTIR Spectral Analysis and Nuclear Magnetic Resonance (NMR) Analysis

ATR-FTIR spectra of SPPs were recorded with an IR Cary 660 FTIR spectrometer (Agilent Technologies, Santa Clara, CA, USA) using a macro-ATR accessory with a Zn/Se crystal. The spectra were measured in a range from 4000 to 500 cm^−1^, with 32 scans both for the background and samples. For all the FTIR analyses, a few mg of sample were used. 

^1^H-NMR and ^13^C-NMR spectra were recorded at 400 and 100 MHz, respectively, on a Bruker AVANCE IIITM 400 spectrometer (Billerica, MA, USA). The obtained proton and carbon shifts (δ) are reported in parts per million related to the residual solvent signal. ^13^C-NMR spectra are ^1^H decoupled. About 5 mg of SPPs was dissolved in 0.75 mL of 99.98% D_2_O (deuterium oxide). 

### 3.4. Monosaccharide Composition by UHPLC-DAD-HR-ESI-Orbitrap/MS Analysis

#### 3.4.1. Hydrolysis of SPPs and Derivatization Procedure

Five mg of SPPs were treated with 1 mL of trifluoroacetic acid (TFA) 4 M, and the mixture was heated at 121 °C for 5 h to hydrolyze the polysaccharide into component monosaccharides. After that, 1 mL of methanol was added, and the TFA residue was removed under vacuum evaporation at 60 °C, and then 150 μL of ultrapure water was added to yield the hydrolyzed solution of polysaccharide ready for the following experiments. PMP derivatization of monosaccharides was carried out as described previously with proper modification [55,56]. Briefly, 150 μL of an aqueous solution of monosaccharide standards (glucose, galactose, mannose, rhamnose, fucose, fructose, xylose, arabinose, glucuronic acid, and galacturonic acid) at 5 mmol/L or hydrolyzed polysaccharides were placed in several 2 mL centrifuge tubes and mixed with 150 μL of NaOH solution 0.6 M and 300 μL of PMP methanol solution 0.5 M for the reaction in a 70 °C water bath for 100 min. Then, 300 μL of HCl 0.3 M were added to the mixture followed by an equal volume of chloroform to remove the PMP. The washing was repeated at least three times. The aqueous samples were centrifuged at 1220 RCF for 10 min, and the supernatant was stored at −20 °C until the analysis.

#### 3.4.2. UHPLC-HR-ESI-Orbitrap/MS Analysis 

The monosaccharide composition of the fraction obtained after hydrolysis of SPPs and derivatization with PMP was analyzed by ultra-high-performance liquid chromatography (UHPLC, Vanquish Flex Binary pump LC) coupled with a diode array detector (DAD, Vanquish HL) and a high-resolution Q Exactive Plus Orbitrap-based mass spectrometer (HR-Orbitrap/MS) equipped with an electrospray ionization source (ESI) (Thermo Fischer Scientific Inc., Darmstadt, Germany). The sample and 10 PMP-labeled monosaccharides/uronic acids used as reference standards were diluted in methanol (1:2), and 5 µL of each solution were injected into the LC-MS system. The elution was carried out on a C-18 Kinetex^®^ Biphenyl column (100 × 2.1 mm, 2.6 μm particle size) provided with a Security Guard^TM^ Ultra Cartridge (Phenomenex, Bologna, Italy), at a flow rate of 0.5 mL/min, using as a mobile phase a mixture of formic acid in H_2_O 0.1% *v*/*v* (solvent A) and formic acid in methanol 0.1% *v*/*v* (solvent B) as follows. The following solvent gradient was applied: 0–3.0 min, 45 to 50% B; 3.0–8.0 min, 50% B isocratic mode; 8.0–9.5 min, 50 to 70% B. The column temperature was maintained at 35 °C. HR mass spectra were acquired in a full (70,000 resolution, 220 ms maximum injection time) and data-dependent MS/MS scan (17,500 resolution, 60 ms maximum injection time), operating in both ESI negative and positive modes (scan range *m*/*z* 400–800). The ionization parameters were optimized as follows: spray voltage, 3400 V (ESI+) and 3200 V (ESI−); capillary temperature, 290 °C; sheath gas (N_2_), 24 arbitrary units; auxiliary gas (N_2_), 5 arbitrary units; HCD (higher-energy C-trap dissociation), 18 eV. DAD data were registered in a range of 200–600 nm, and 250 nm was selected as a preferential channel for the detection of PMP-derivatives. Xcalibur 4.1 software was used for data processing.

### 3.5. Radical Scavenging Assays

#### 3.5.1. ABTS Radical Scavenging Assay

ABTS radical scavenging activity was determined according to the method previously described by Li et al. [57]. The ABTS^•^ stock solution was produced by reacting 10 ml of 7 mM ABTS aqueous solution and 163 μL of 150 mM (2.45 mM final concentration) potassium persulfate solution. After storage for 16 h at 4 °C in the dark, this solution was diluted in ethanol until an absorbance of 0.72 ± 0.01 (at 734 nm) was reached. Then, 180 µL of ABTS^•^ solution was mixed with 20 µL of solution of SPPs in water/DMSO 4:1 at different concentrations (from to 0.025 mg/mL to 1.0 mg/mL) in 96-well plates. The obtained mixture was incubated for 15 min at room temperature in darkness. The neutralization of the ABTS radical leads to a decrease in absorbance monitored against a water/DMSO (1:4) mixture as a blank at 734 nm using an EnSpire UV-Vis spectrophotometer. (Thermo Fisher Scientific, Monza, Italy). The control was prepared by mixing 180 µL of ABTS^•^ solution with 20 µL of water/DMSO (1:4). The absorbance measured for the control solution was in the range of 0.720. The percentage results of scavenging effects were calculated as % inhibition using the following equation:

$$\%\mathrm{inhibition}=\frac{\left(\;\mathrm{Abs}\;\mathrm{control}\;-\;\mathrm{Abs}\;\mathrm{sample}\;\right)}{\;\mathrm{Abs}\;\mathrm{control}\;}\times 100$$

#### 3.5.2. Hydroxyl Radical (^•^OH) Scavenging Assay

The ^•^OH scavenging activity was determined according to the method described by Chen et al. with some modification [58]. Briefly, 50 µL of 6.0 mM FeSO_4_ and 50 µL of 6.0 mM ethanol salicylate were mixed with 50 µL of a solution of SPPs in water/DMSO 4:1 at different concentrations (from 0.08 mg/mL to 2.5 mg/mL). After that, 50 µL of 6.0 mM H_2_O_2_ were added to start the reactions, and the samples were incubated at 37 °C for 30 min. The Fenton reaction produces ^•^OH which reacts with the salicylate solution. The neutralization of the ^•^OH radical was monitored against a water/DMSO (1:4) mixture as a blank at 510 nm using an EnSpire UV-Vis spectrophotometer. The control was prepared by mixing 50 µL of water/DMSO (1:4) with 50 µL of FeSO_4_ solution, 50 µL of ethanol salicylate solution, and 50 µL of H_2_O_2_. The absorbance measured for the control solution was in the range of 1.00. The radical inhibition percentage was calculated by application of the equation reported above.

Ascorbic acid was used as a positive control, and all the experiments were conducted in triplicate. The EC50 values were calculated using the statistical program GraphPad Prism^®^ version 10 (GraphPad Software Inc., San Diego, CA, USA). 

### 3.6. Cell Culture

HEI-OC1 (House Ear Institute-Organ of Corti 1) cells were maintained in high-glucose Dulbecco’s modified Eagle’s medium (DMEM) supplemented with 10% fetal bovine serum and 2.5 μg/mL amphotericin B at 33 °C in a humidified incubator with 10% CO_2_, as described in [59]. Amphotericin B was obtained from Sigma/Merck (Darmstadt, Germany); DMEM and fetal bovine serum were purchased from Euroclone (Milan, Italy).

### 3.7. Viability Experiments

Five thousand HEI-OC1 cells were seeded in 96-well plates containing 100 µL of medium. The following day, cells were treated in the presence or the absence of different concentrations of SPPs and 5 μM cisplatin. After 48 h, cell viability was measured evaluating 3-(4,5-dimethylthiazol-2-yl)-2,5-diphenyltetrazolium bromide (MTT) reduction. Briefly, 0.5 mg/mL MTT in phosphate buffer saline was added to each well; cells were incubated at 33 °C in a humidified 10% CO_2_/90% air atmosphere for 60 min; the reaction was stopped by adding 175 µL of dimethyl sulfoxide, and the formazan salts were dissolved by gentle shaking for 60 min at 37 °C and quantified spectrophotometrically by reading the absorbance at 596 nm with an automatic ultra-microplate reader, EL 808 (Bio-Tek Instruments Inc., Winooski, VT, USA). The values were normalized against the average absorbance of the control sample. The results were expressed as means ± SEM from 2–4 experiments performed in octuplicate.

### 3.8. Evaluation of Intracellular Reactive Oxygen Species (ROS)

The intracellular ROS level was measured by using DCFH-DA, which is converted to fluorescent 2′,7′-dichlorofluorescein in the presence of an oxidant. For the assay, HEI-OC1 cells (2 × 10^4^ cells/well) were plated in 96-well black plates, and, the following day, they were treated in the presence or the absence of 30 µM cisplatin and an active concentration of SPPs, i.e., 80 μg/mL for 4 h. The cell medium was removed, and the cells were incubated in the dark for 30 min at 37 °C in Hank’s solution containing 5 µM DCFH-DA. The emitted fluorescence was measured every 2 min for 60 min with a FluoSTAR OPTIMA microplate reader (BMG LabTech GmbH, Ortenberg, Germany) at 485 nm excitation and 520 nm emission. The variation in fluorescence versus time in each well was normalized with the viability measured by the MTT assay. The results were further normalized vs. the control values and were expressed as means ± SEM from 2 experiments performed in triplicate.

### 3.9. Statistical Analysis

Data are presented as means ± SEM of the respective n values (Prism 9; GraphPad software, San Diego, CA, USA). The results are compared using ANOVA followed by Tukey’s multiple comparisons test. A value of *p* < 0.05 was considered significant.

## 4. Conclusions

The search for new agents able to reduce the cisplatin-induced damage to the ear is certainly of great interest, in order to make the use of this oncological drug safer.

In agreement with the antioxidant properties described for microalgal and cyanobacterial polysaccharides [28], and confirmed in the present work both in spectrophotometric radical scavenging assays and in the cochlear cell model, for the SPPs extract, a significant protective activity on the reduction in viability induced by the chemotherapy agent was obtained. This protection occurred even when the polysaccharides were preincubated and not co-administered with cisplatin, allowing for the exclusion of the conclusion that the polysaccharides acted by reducing the entry of cisplatin into the cell. A potential linkage among the sulfate groups (negative charged) and the cisplatin molecule after its loss of chloride anions due to the aqueous cell environment [60] was in fact hypothesized.

In conclusion, our data suggest that the natural carbohydrate polymers can be used to prevent or counteract ototoxicity from cisplatin or other ototoxic agents with activity similar to cisplatin.

The possible use in vivo opens up, as expected, the challenge of the bioavailability at the inner ear, since the cochlea is among the most difficult organs to reach after conventional administration routes. Moreover, another problem is linked to the possible interference with the cytotoxic activity of cisplatin specifically on cancerous tissues. Local administration using strategies crossing the tight junction-coupled blood–labyrinth barrier (BLB), such as trans-tympanic (intra-tympanic) delivery or intracochlear application, is surely a way to overcome both of these challenges [61]. Supporting this, several papers already reported efficacy in clinical trials with molecules undergoing trans-tympanic (intra-tympanic) administration for contrasting cisplatin ototoxicity [9,10,62,63]. Topical innovative drug delivery systems are currently under investigation, among which are hydrogels [61]. In this regard, it is relevant that natural polysaccharides may be a useful biomaterial for realizing biocompatible hydrogels for drug delivery strategies. In the case of microalgal/macroalgal polysaccharides, our study suggests the possibility to create a delivery system that is not only biocompatible but also otoprotective in itself.

## Figures and Tables

**Table 1:** Chromatographic, UV, and mass spectral data of 1-phenyl-3-methyl-5-pyrazolone (PMP)-labeled monosaccharide detected in the hydrolyzed SPPs.

Peak ^a^	Compound	t_R_ (min)	HR-[M − H]^−^ (*m*/*z*)	Product Ions (*m*/*z*) ^c^	HR-[M + H]^+^ (*m*/*z*)	Product Ions (*m*/*z*) ^c^	Molecular Formula	Error (ppm) ^d^
1	Rhamnose-PMP ^b^	3.1	493.2098	**215.08**, 173.07, 92.05	495.2231	373.17, 217.10, **175.09**	C_26_H_29_N_4_O_6_	+1.014
2	Ribose-PMP ^b^	3.2	479.1939	**215.08**, 173.07, 92.05	481.2075	373.17, 217.10, **175.09**	C_25_H_28_N_4_O_6_	+0.626
3	Glucose/galactose/fructose-PMP ^b^	4.8	509.2043	**215.08**, 173.07, 92.05	511.2180	373.17, 217.10, **175.09**	C_26_H_30_N_4_O_7_	−0.196
4	Pentose-PMP	5.3	479.1939	**215.08**, 173.07, 92.05	481.2075	373.17, 217.10, **175.09**	C_25_H_28_N_4_O_6_	+0.626
5	Fucose-PMP ^b^	5.7	493.2098	**215.08**, 173.07, 92.05	495.2231	373.17, 217.10, **175.09**	C_26_H_29_N_4_O_6_	+1.014

^a^ Peak numbers correspond to those of Figure 3. ^b^ Confirmed by reference standard. ^c^ The base ion peaks are shown in bold. ^d^ Calculated on [M − H]^−^ ion.

## Data Availability

Data is contained within the article.
